# CO_2_ Sensing Using Symmetrical Three-Wavelength Precompensated Current-Modulated Tunable Diode Laser Absorption Spectroscopy

**DOI:** 10.3390/s26051420

**Published:** 2026-02-24

**Authors:** Giacomo Zanetti, Peter John Rodrigo, Christian Pedersen

**Affiliations:** DTU Electro, Department of Electrical and Photonics Engineering, Technical University of Denmark, Frederiksborgvej 399, Building 128, 4000 Roskilde, Denmark; pejr@dtu.dk (P.J.R.); chrp@dtu.dk (C.P.)

**Keywords:** CO_2_ remote sensing, gas spectroscopy, tunable diode laser absorption spectroscopy, diode laser frequency modulation, differential absorption lidar

## Abstract

**Highlights:**

A third, symmetrically placed wavelength was added to the differential absorption spectroscopy archetype to obviate baseline instability when remotely measuring CO_2_ concentrations. An appropriate current shaping was used to offset the diode laser’s slowest time constants, achieving an update rate of 2 kHz with a single laser source. The method was compared to the standard two-wavelength differential absorption spectroscopy approach.

**What are the main findings?**

- Fourfold improvement in stability over 24 days.
- Eight times weaker correlation between concentration and temperature.

**What are the implications of the main findings?**

- Substantial improvement in long-term stability with matched short-term performance.
- Demonstrated a reduced effect of baseline instability on concentration measurements.

**Abstract:**

In this paper, a novel symmetrical three-wavelength toggling archetype for measuring the concentration of gases using a tunable diode laser absorption spectroscopy (TDLAS) system is introduced and demonstrated. The system was operated at 1.5714 µm with a 2 kHz update rate, targeting an absorption line of gaseous CO_2_. Precompensated diode–current pulses are introduced to offset the inherent thermal time constants of the diode laser by orders of magnitude. Here, repetition rates matching that of contemporary methods can be achieved, while simultaneously providing a noteworthy wavelength stability of 0.6 pm for the three targeted wavelengths that are approximately 70 pm apart (142 pm maximum wavelength excursion). A 10 Hz current loop locks one of the wavelengths to a CO_2_ absorption peak, thus providing an absolute and stable wavelength reference. The flexibility in choosing the shape and repetition frequency of the current pulses makes this approach easily adaptable to other gases and/or absorption lines, since wavelength filters are avoided. The new method is benchmarked against a two-wavelength precompensated continuous-wave TDLAS technique, revealing a fourfold improvement in reproducibility with system restart over the span of 24 days, while outperforming other widespread spectroscopic techniques applied to comparable transmittance levels. The effect of the analytical model was further studied by thermally inducing baseline changes, showing a 7.9 ± 0.2 times weaker correlation between concentration and temperature with respect to the one observed using the two-wavelength TDLAS archetype. These results demonstrate the system’s suitability for sensitive applications.

## 1. Introduction

Being able to precisely change the wavelength of a laser source in the microsecond-to-millisecond timescale is a requirement present in an exceptionally broad range of applications, from the reconfiguration of telecommunication sessions [1] all the way to wavelength-based beam steering [2,3,4] and laser-based detection and ranging (lidar) [4]. Among these, remote detection and quantification of gases is an important example which can be found in various fields, from workplace safety [5,6,7] to product quality control, and from the surveillance and regulation of emissions to the detection of chemical warfare agents [8]. In some of these contexts, real-time measurements can have a pivotal role, as they may serve as an alarm or visualization tool for operators. A solution to this is differential absorption lidar (DIAL), a spectroscopic technique where laser(s) transmit two or more distinct wavelengths through the atmosphere to probe the absorption of the targeted gases. These wavelengths are usually kept close to each other in order to neglect the wavelength dependency of aerosols, optical components and, more generally, all wavelength-dependent effects besides the molecular absorption of the gases. On the other hand, two wavelengths are sufficient to quantify up to a single gas species; hence, the DIAL technique is typically employed to target a specific gas, such as H_2_O, NO_2_, SO_2_ or O_3_. Despite this, research has shown that utilizing three wavelengths instead of two can be beneficial even when trying to detect a single gas [9,10,11]. In such studies, the third wavelength was used either to probe additional gas lines of the same gas species or to reduce the effect of ozone and aerosol absorption by minimizing and/or maximizing wavelength-dependent analytical parameters connected to the collective absorption of the gases in the atmosphere.

In this paper, we introduce a new approach where the third wavelength is used to mitigate the instability of the absorption baseline, namely the absorption spectrum along the light’s path in the absence of the targeted gas. This method is demonstrated by extending our novel tunable diode laser absorption spectroscopy (TDLAS) system for experiments that exploit the differential absorption spectroscopy principle under continuous-wave operation [12,13], rather than with the conventional pulsed lasers used in DIAL. In fact, our TDLAS system employs a single tunable laser that is quickly toggled between two wavelengths. This is achieved by precompensating the current modulation of the laser, enabling transitions on a timescale approximately two orders of magnitude shorter than dictated by the thermal constants of the laser’s response [14], which allows the system to reach update rates of up to 8 kHz. We refer to this as two-wavelength TDLAS. Despite the impressive speed this method can achieve, its analytical simplicity makes it prone to errors coming from the time-varying optical characteristics of the components used, which can change due to, e.g., temperature, alignment, or aging. In fact, although two-wavelength TDLAS is a calibration-free technique, meaning that it does not need external knowledge of concentration values to make an absolute concentration measurement, it is widely known that the optical properties of the components present in the setup can change enough to invalidate initial baseline calibrations, imposing severe limitations on the system’s stability [15]. A solution is to use a high number of wavelengths to model the baseline [16], which greatly increases system complexity and computational requirements. In response to this, we hereby develop and demonstrate a near-infrared (NIR) system based on three-wavelength TDLAS for CO_2_ quantification, showing how it partially obviates the baseline instability problem without increasing computational requirements. We benchmark the performance of the new method against that of the two-wavelength TDLAS approach at a 2 kHz update rate, with particular interest in stability over time and reproducibility after system restart.

## 2. Theoretical Framework

In the absence of any absorbing gas species, the transmittance spectrum around the laser’s wavelength is generally not flat due to the wavelength-dependent optical properties of the optical elements used, such as beamsplitters, polarizers, waveplates and cavities. We refer to this transmittance spectrum as “transmittance baseline”, $T_{b}\left(\delta \nu \right)$, where δν is the radiation’s detuning with respect to the center of the probed absorption line. In the presence of a gas, the measured transmittance $T\left(\delta \nu \right)$ is affected by the absorption of the gas as described by Lambert–Beer’s law of extinction, namely $T\left(\delta \nu \right)=T_{b}\left(\delta \nu \right)\cdot e^{-\alpha \left(\delta \nu \right)}$, where $\alpha \left(\delta \nu \right)$ is the gas’ absorption coefficient. Two-wavelength TDLAS is based on the DIAL equation

(1) $$n=\frac{1}{2\Delta R\left(\sigma \left(0\right)-\sigma \left(\delta \nu_{OFF}\right)\right)}\;\ln\left(\frac{P_{off}\left(R_{2}\right)}{P_{on}\left(R_{2}\right)}\frac{P_{on}\left(R_{1}\right)}{P_{off}\left(R_{1}\right)}\right)$$

where n represents concentration, $R_{1},R_{2}$ are the initial and final ranges of the probed interval of length $\Delta R=R_{2}-R_{1}$, σ is the absorption cross section of the gas, and $P_{on},P_{off}$ are the measured on- and off-resonance powers, respectively. Note that the factor 2 in the denominator assumes that the collected power is backscattered and thus that light has crossed the same path twice before being detected, while the absorption coefficient α is connected to the absorption cross-section and path length as α=σΔR. Our two-wavelength TDLAS approach operates in the particular case where a reference detector is used, namely when $R_{1}=0$ and $\Delta R=R_{2}$. Therefore, the calculation of the gas’ concentration is performed by taking the negative logarithm of the transmittance at the two wavelengths. Using the previous definitions, for two-wavelength TDLAS one calculates

(2) $$-\ln\left(\frac{T_{ON}}{T_{OFF}}\right)=-\ln\left(\frac{T_{b}\left(0\right)}{T_{b}\left(\delta \nu_{OFF}\right)}\right)+\alpha \left(0\right)-\alpha \left(\delta \nu_{OFF}\right)\;$$

where δν=0 for the on-resonance wavelength and $\delta \nu_{OFF}=\nu_{OFF}-\nu_{ON}$. From here, libraries such as HITRAN [17] can be used to derive concentration by explicitly expressing the dependence of α on it. The caveat is that while the left-hand side of the equation is obtained experimentally, the baseline is not typically monitored and, in some cases, it could be simply impossible to do so (e.g., the gas cannot be removed after the start of the experiment). Therefore, any change in the ratio $\frac{T_{b}\left(0\right)}{T_{b}\left(\delta \nu_{OFF}\right)}$ would be mistaken for a change in concentration. On the other hand, this method is immune to strictly proportional changes in the baseline which can be caused by the introduction or removal of filters or attenuators, variable losses due to aerosol, a moving target, slight misalignments due to vibrations or thermal expansion/contraction of the mounts and, in general, anything that has an impact on transmission with a negligible wavelength dependency on the scale on which the toggling happens (well below the nanometer scale for the NIR CO_2_ lines used in this study). Beyond this, having a flat baseline also filters out any rigid translation, as the ratio $\frac{T_{b}\left(0\right)}{T_{b}\left(\delta \nu_{OFF}\right)}$ would remain constant. Despite this robustness, such a protocol is still affected by changes in the optical properties of the optical components used, especially those that rely on interference (e.g., beamsplitters, cavities), as their behavior is extremely susceptible to refractive index changes and physical deformation. In our experiments, due to the very thin spectral width of the probed absorption lines, such changes are mostly driven by temperature and usually manifest themselves as linear tilts of the baseline. Although small relative to the transmittance, these tilts are big enough to cause changes in the calculated concentration values that exceed the short-term (i.e., the timescale over which the baseline does not change) repeatability of the system. Therefore, a more robust method to derive gas concentrations is needed when baseline tilting is a relevant phenomenon and baseline characterization is unfeasible or expensive.

Like any other function, the transmittance baseline function $T_{b}\left(\delta \nu \right)$ can be separated into its even and odd parts, respectively $E_{b}\left(\delta \nu \right)$ and$\;O_{b}\left(\delta \nu \right)$, as

(3) $$T_{b}\left(\delta \nu \right)=\frac{T_{b}\left(\delta \nu \right)+T_{b}\left(-\delta \nu \right)}{2}+\frac{T_{b}\left(\delta \nu \right)-T_{b}\left(-\delta \nu \right)}{2}=E_{b}\left(\delta \nu \right)+O_{b}\left(\delta \nu \right)$$

with the implication that $E_{b}\left(0\right)=T_{b}\left(0\right)$ and $O_{b}\left(0\right)=0$. From their definitions, it follows that the computation of $E_{b}$ and $O_{b}$ requires the knowledge of the transmittance at both +δν and −δν, meaning that two off-resonance frequencies, symmetrically placed about the absorption line, must be probed. Additionally, if the radiation used lies in a spectral region where only the probed gas absorbs, and the neighboring absorption peaks are far enough to be neglected in the calculation of $\alpha \left(\delta \nu \right)$, then $\alpha \left(\delta \nu \right)=\alpha \left(-\delta \nu \right);$ we refer to this approximation as “isolated peak approximation”. Under this approximation, the experimental measurements of $T\left(\delta \nu \right)$ and $T\left(-\delta \nu \right)$ can be written as $T_{exp}\left(\delta \nu \right)=T_{b}\left(\delta \nu \right)\cdot e^{-\alpha \left(\delta \nu \right)}$ and $T_{exp}\left(-\delta \nu \right)=T_{b}\left(-\delta \nu \right)\cdot e^{-\alpha \left(\delta \nu \right)}$, respectively. Therefore, explicating $T\left(\delta \nu \right)$ gives

(4) $$T\left(\delta \nu \right)=T_{b}\left(\delta \nu \right)\cdot e^{-\alpha \left(\delta \nu \right)}=E_{b}\left(\delta \nu \right)\cdot e^{-\alpha \left(\delta \nu \right)}+O_{b}\left(\delta \nu \right)\cdot e^{-\alpha \left(\delta \nu \right)}=E\left(\delta \nu \right)+O\left(\delta \nu \right)$$

(5) $$E\left(\delta \nu \right)=E_{b}\left(\delta \nu \right)\cdot e^{-\alpha \left(\delta \nu \right)}=\frac{T_{exp}\left(\delta \nu \right)+T_{exp}\left(-\delta \nu \right)}{2}$$

(6) $$O\left(\delta \nu \right)=O_{b}\left(\delta \nu \right)\cdot e^{-\alpha \left(\delta \nu \right)}=\frac{T_{exp}\left(\delta \nu \right)-T_{exp}\left(-\delta \nu \right)}{2}$$

where E and O are, respectively, even and odd functions because the symmetry of $e^{-\alpha \left(\delta \nu \right)}$ preserves the parity of $E_{b}$ and $O_{b}$.

Replacing $T(\delta \nu_{OFF})$ with any other measurable quantity that depends on concentration through $\alpha \left(\delta \nu_{OFF}\right)$ according to Lambert–Beer’s extinction law does not change the functional form of Equation (2). In particular, $E\left(\delta \nu \right)$ satisfies this condition and, contrary to the generic shape of $T\left(\delta \nu \right)$, is strictly even. This means that any odd component of the baseline, such as a linear tilt, does not affect $E\left(\delta \nu \right)$ and, consequently, has no impact on the calculation of the gas’ concentration. Conversely, even terms have the same effect they would have on $T\left(\delta \nu \right)$. Moreover, just as in the case where $T(\delta \nu_{OFF})$ is used, a proportional change in $T_{b}$, as well as rigid translations in the case of a flat baseline, do not affect concentration. Therefore, at the cost of introducing another off-resonance wavelength, by replacing $T\left(\delta \nu \right)$ with $E\left(\delta \nu \right)$ one gets a system that is much more robust against baseline changes over time. Moreover, due to the functional simplicity of $E\left(\delta \nu \right)$, using it in place of $T\left(\delta \nu \right)$ does not relevantly increase computational requirements.

A similar discussion could be made for $O\left(\delta \nu \right)$. However, using $O\left(\delta \nu \right)$ prevents operation with an initially flat baseline and has a probability of going to zero, leading to a singularity in Equation (2). This, combined with the experimental observations that prove the presence of linear tilts in the baseline, makes it a much less attractive candidate.

Because this discussion is based on the isolated peak approximation, particular care must be taken when deciding the parameters of the current modulation. In fact, there is a trade-off between the adequacy of this approximation and the stability offered by having the off-resonance wavelengths away from the peak, ideally in a spectroscopically flat region where the laser’s wavelength instability is less relevant. Another aspect of interest is the optimization of the distribution of the available datapoints between the different wavelengths.

Lastly, the three-wavelength TDLAS method outlined here can be generalized to asymmetrical absorption lines, although the benefits vary with both the extent of the asymmetry and the functional form of the baseline—that is, whether it is predominantly odd or even. This is done by imposing $\alpha \left(\delta \nu_{1}\right)=\alpha \left(\delta \nu_{2}\right)$ without the constraint $\delta \nu_{1}=-\delta \nu_{2}$, which preserves the functional form of Equation (2). In this case, though, the separation of $T\left(\delta \nu \right)$ as per Equations (4)–(6) is no longer exact and the suppression of the effect of baseline fluctuations depends on how much the choice of off-resonance wavelengths deviates from the ideal case $\delta \nu_{1}=-\delta \nu_{2}$. Otherwise, the method can be applied to any asymmetrical line if the central frequency is shifted from resonance so to satisfy $\delta \nu_{1}=-\delta \nu_{2}$, with $\alpha \left(\delta \nu_{1}\right)=\alpha \left(\delta \nu_{2}\right)$, though it can come at the cost of a reduced signal-to-noise ratio and other practical complications.

## 3. Materials and Methods

The setup used was the same as the one described in [13] and consists of a 1.571 µm diode laser (EP1572-5-NLW-B26-100FM, Eblana Photonics, Dublin, Ireland) which is fiber-coupled to a polarization-maintaining fiber of about 1 m in length, at the end of which is a beam collimator (F220FC-1550, Thorlabs Inc., Newton, NJ, USA). The laser is driven by a laser diode current controller (LDC205C, Thorlabs Inc., Newton, NJ, USA), while its temperature is regulated by a temperature controller (TED 200, Thorlabs Inc., Newton, NJ, USA).

**Figure 1:**
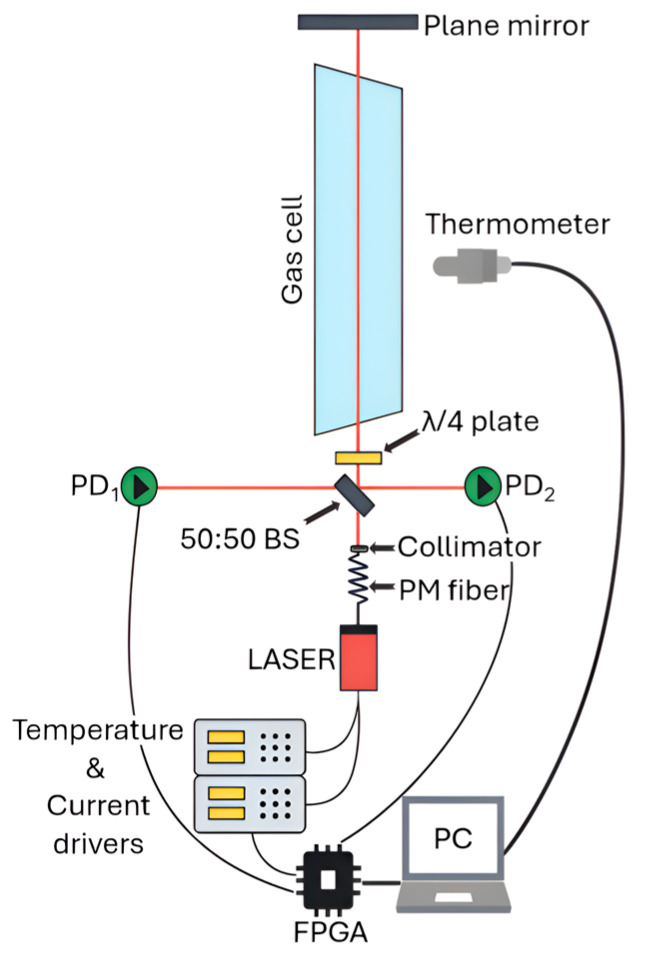
Sketch of the experimental setup. The green circles represent the photodetectors (PD), namely PD_1_ acting as reference and PD_2_ as the gas arm.

After the collimator, the laser beam propagates in free space through a 50:50 non-polarizing plate beamsplitter (10B20NP.31, Newport Corporation, Irvine, CA, USA) after which one beam is detected by a first InGaAs photodiode detector (FGA21, Thorlabs Inc., Newton, NJ, USA), which acts as reference (PD_1_ in Figure 1). The second beam passes a λ/4 achromatic waveplate (A-12.7-A-.250-B-3, Edmund Optics Inc., Barrington, NJ, USA) and then goes through a 40 cm custom-made gas cell (Wavelength References Inc., Corvallis, OR, USA) filled with a mixture of C_2_H_2_/CH_4_/CO_2_/N_2_. The nominal volume mixing ratio is 1% C_2_H_2_, 3% CH_4_, 80% CO_2_, and the cell is balanced with N_2_ to 740 Torr total pressure. The gas cell has tilted wedged windows made of B270 Glass AR-coated for 1550 nm. A plane mirror is used to trace the laser beam back to the beamsplitter, which then directs part of the radiation towards a second photodetector (PD_2_ in Figure 1, nominally identical to PD_1_), which serves as the gas arm. Given the ~5 cm optical path between the different components of the setup, the high CO_2_ concentration in the gas cell, and the negligible absorption from other gases, the probed range Δ*R* effectively corresponded to the length of the cell (see Equation (1)).

Most of the electronic components are controlled by a field-programmable gate array (FPGA) embedded in a controller (cRIO-9063, National Instruments, Austin, TX, USA) which mounts a digital-to-analog converter (NI-9263, National Instruments, Austin, TX, USA) and an analog-to-digital converter (NI-9223, National Instruments, Austin, TX, USA). The 128 kSamples/s sampling rate, combined with 64 points per period, determines the 2 kHz update rate. Lastly, a laptop computer (EliteBook 840 G10, HP Inc., Palo Alto, CA, USA) is used to retrieve and elaborate the FPGA readings through a LabVIEW 2024 Q3 (64-bit) (National Instruments, Austin, TX, USA) self-developed code, as well as to control a temperature sensor (TSP01, Thorlabs Inc., Newton, NJ, USA) positioned in the gas cell’s proximity, whose output was read every 3 s. The gas cell is assumed to be at thermal equilibrium with the room.

**Figure 2:**
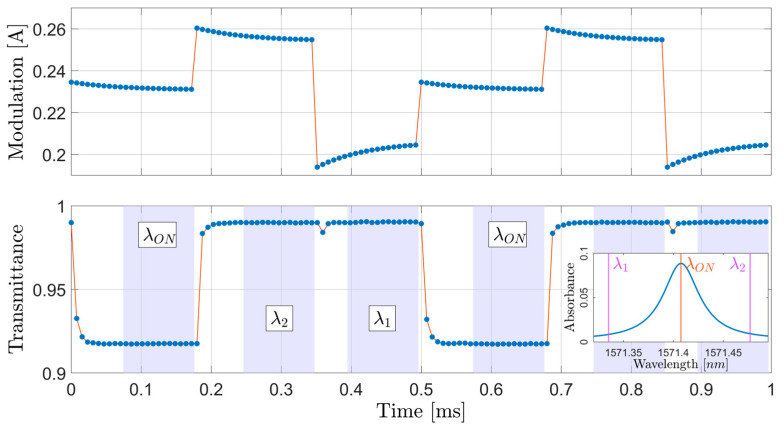
Laser diode current controller’s output and normalized transmittance over two $\lambda_{1}$-$\lambda_{ON}$-$\lambda_{2}$ sequences. The inset shows the location of the wavelengths used with respect to the absorbance profile of CO_2_ around 1571.4 nm at our typical experimental conditions, namely a temperature of 295.12 K and pressure of 740 Torr.

A precompensated laser current modulation was applied to achieve fast transitions between wavelengths, transitions that occurred in growing order starting from the first off-resonance wavelength ($\lambda_{1}$), then to the on-resonance one ($\lambda_{ON}$) and finally to the second off-resonance wavelength ($\lambda_{2}$). This sequence constitutes a period, meaning that one concentration value was calculated for each $\lambda_{1}$-$\lambda_{ON}$-$\lambda_{2}$ sequence. Figure 2 illustrates the laser diode current controller’s output used to achieve this three-wavelength sequence, together with the normalized transmittance data that are processed in real-time to calculate the concentration of CO_2_ in the gas cell.

As the variance of the on-resonance transmittance is not affected, to the first order, by wavelength uncertainty due to a null $\frac{dT}{d\lambda }$, subtracting it from the noisy $\lambda_{1}$ transmittance can isolate the effect of the wavelength instability on transmittance. Assuming that the non-wavelength sources of error are equal for the on-resonance and off-resonance cases, this calculation yields a standard uncertainty of 0.6 pm. This means that wavelength transitions in the order of 140 pm (17 GHz) can be achieved within approximately 50 µs with a relative uncertainty of about 0.5% by correctly precompensating the current modulation of the laser; without doing it, the transitional times would be of several milliseconds, as shown in [12].

The duty cycle was split into 30%, 35% and 35% for $\lambda_{1}$, $\lambda_{ON}$ and $\lambda_{2}$, respectively. This distribution was chosen because it allowed for the fastest transition between all three wavelengths, though it should not be understood as a rule to follow when employing this method. Furthermore, the amplitude of the modulation, hence the separation between wavelengths, was determined by compromising its maximization (to ensure a high signal-to-noise ratio) with the transition times between wavelengths and with the increased noise caused by larger current modulations. It is also worth noting that virtually any wavelength within the laser’s dynamic range can be targeted by setting the proper modulation parameters, which gives the proposed method enough flexibility to be applied for other gases and/or absorption lines.

**Figure 3:**
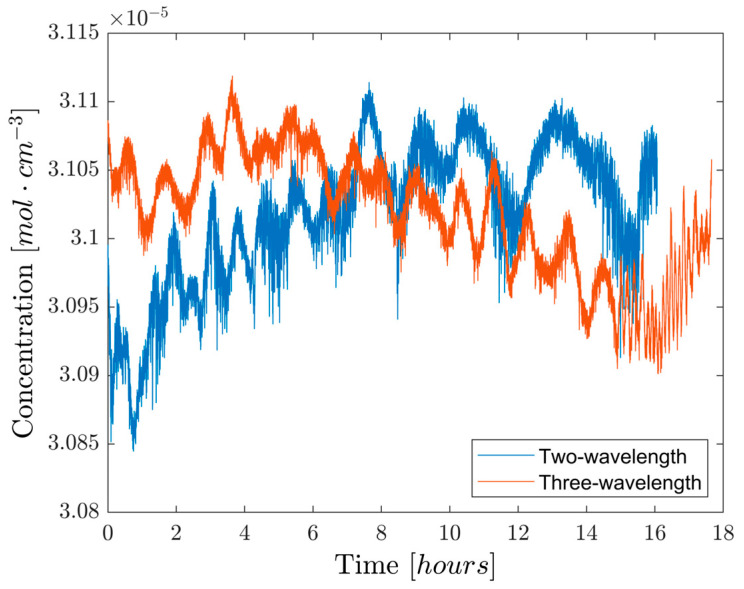
Overnight concentration measurements. The three-wavelength method’s results are calibrated using the previously recorded two-wavelength data shown in [13]. Both datasets are subject to a factor 200 decimation. The nominal concentration of the gas cell is (3.13 ± 0.16) × $10^{-5}$ mol/cm^3^.

The $\lambda_{ON}$ was kept on-resonance using a hill-descent algorithm working at an update time of 100 ms. In detail, the algorithm consisted in changing the current in 5 µA steps (positive or negative) and checking the measured change in the on-resonance transmittance, low-pass filtered at 300 Hz: if the current change caused the transmittance to increase, the next current step would have been of the opposite sign, otherwise the same. To limit the effect of small fluctuations in the photodiodes’ readings, happening on a timescale of about 1 s, a threshold of 10 same-sign steps was given, after which the step was negated. Due to the non-stationarity of the system over extended periods of time and temperature/pressure changes, an algorithm without definitive stopping criteria was necessary. The same algorithm was used in [13]. The absolute value of the $\lambda_{1}$ wavelength was then fixed by calibrating the readouts with the 2 kHz two-wavelength TDLAS overnight results obtained in [13] and shown in Figure 3. The other off-resonance wavelength was set by experimentally ensuring baseline flatness and then finding the current modulation that made the $\lambda_{1}$ and $\lambda_{2}$ transmittance values compatible. Under the assumption that the absorption line is isolated, this procedure ensures symmetrical frequency values around the center of the peak for the two off-resonance wavelengths.

Having one more transition per period reduces the number of usable points. In fact, compared to the 42 points used in two-wavelength TDLAS, only 39 out of the 64 available points were used in the three-wavelength method (13 points each at $\lambda_{1}$, $\lambda_{ON}$ and $\lambda_{2}$), a 7% decrease in data usage efficiency. When operating the system in the three-wavelength TDLAS mode, the temperature of the laser was raised from 25.00 °C to 28.76 °C to probe the 1.5714 µm absorption line, which is closer to being symmetrical compared to the 1.5711 µm line used in [13] because of the position of other weaker CO_2_ absorptions lines.

## 4. Results and Discussion

In Figure 3, an overnight measurement made with the three-wavelength TDLAS method is compared with the two-wavelength TDLAS result shown in [13], which also served to calibrate the former. Despite the lower number of points per cycle used, the relative maximum excursion of the data is 0.70%, lower than the 0.87% observed in the two-wavelength approach. While both measurements show fluctuations in the hour timescale, the readings made with three-wavelength TDLAS are characterized by a standard deviation of 0.14%, compared to the 0.17% of two-wavelength TDLAS. Both metrics hint at an improvement in long-term stability when using three-wavelength TDLAS over its two-wavelength counterpart.

It can nonetheless be seen that the aforementioned fluctuations increase in frequency after the fifteenth hour in the three-wavelength TDLAS experiment. This is most likely due to the recorded sudden and fast increase in temperature in the laboratory caused by the sunrise, which changed the optical properties of the optical components in the setup. Such an observation suggests that, despite correcting for linear baseline tilts, using three wavelengths is still not enough to completely obviate the changes in the components’ optical properties caused by room temperature variations, about 2 °C for an overnight experiment. Therefore, active temperature control of the components is needed even when using this method, although the requirements on its precision are likely lower than in the two-wavelength case due to the reduction in the baseline’s influence on concentration.

**Figure 4:**
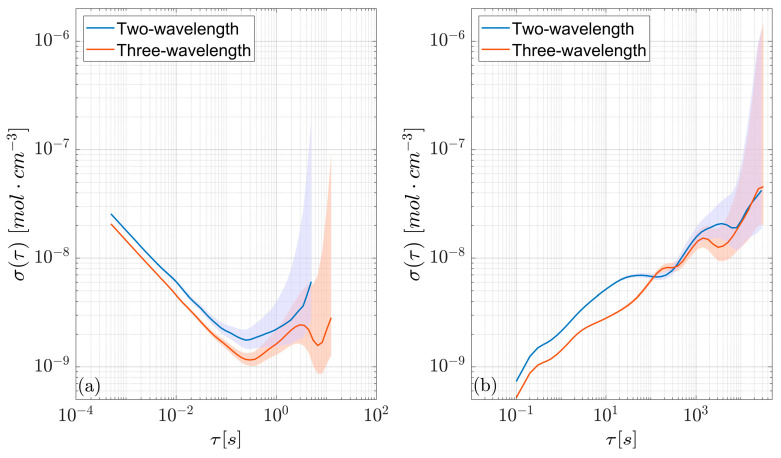
Allan deviations of the two- and three-wavelength TDLAS approaches. The short-timescale values (**a**) were calculated from minute-long experiments, whereas the long-timescale values (**b**) were computed using the decimated datasets shown in Figure 3. Shaded areas represent the 95% confidence interval.

To cover a broad range of integration times, the non-overlapping Allan deviations of the two methods were calculated for minute-long experiments (Figure 4a) as well as for measurements lasting several hours (Figure 4b). For the longer measurements, the data acquisition was decimated by factor 200 to reduce storage requirements. Comparing the non-overlapping Allan deviations shown in Figure 4 reveals that three-wavelength TDLAS performed better up to an integration time τ of about 100 s, which is surprising due to the lower number of points per period used. In this regime (τ<100 s) there are no conditions where two-wavelength TDLAS should be preferred over its three-wavelength counterpart. Moreover, both Allan deviations reach their lowest values at a remarkably similar timescale, about 0.25 s. Consequently, the minima of the Allan deviations portrayed in Figure 4b occur at the first integration times for which the quantity can be calculated. The two methods perform comparably on longer timescales, but the uncertainty on the Allan deviation makes it hard to draw definitive conclusions. To put the obtained results into perspective, the minimum relative Allan deviation observed here is almost an order of magnitude smaller than that obtained from absorptions of similar intensity using other widespread methods such as wavelength-scanned TDLAS, wavelength modulation spectroscopy (WMS) and heterodyne phase-sensitive dispersion spectroscopy (HPSDS), although they rely on more convoluted architectures and data processing [18].

Resetting the instrument may cause slight yet meaningful changes in its working point, which is extremely relevant when determining the reliability of a device. Therefore, the repeatability with system restart was investigated by performing a 1 s acquisition every two minutes. This experiment showed that three-wavelength TDLAS, in these conditions, performed better than its two-wavelength counterpart. The maximum absolute relative difference in concentration observed between subsequent cycles was 0.13% (mean 0.06 ± 0.01%), compared to the 0.30% (mean 0.08 ± 0.03%) of two-wavelength TDLAS. Although the mean values are compatible (*p* = 0.52), the smaller standard error on the three-wavelength figure highlights that this method is less likely to give substantial deviations (e.g., >0.1%) between results of successive experiments.

**Figure 5:**
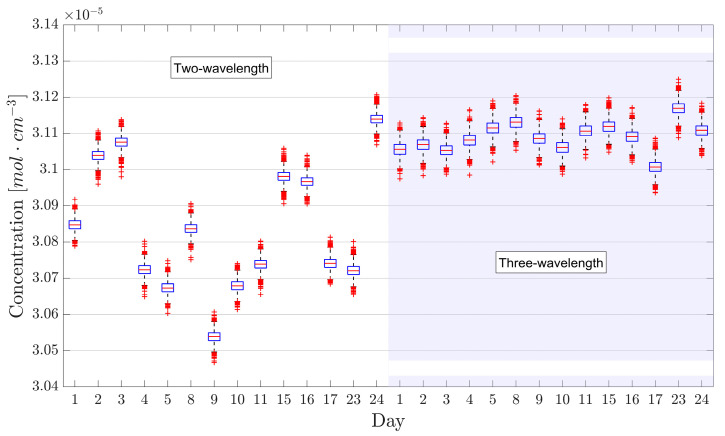
Daily concentrations measurements made with two-wavelength TDLAS (**left half**) and three-wavelength TDLAS (**right half**). The blue boxes represent the 25th and 75th percentiles, the orange horizontal lines are the medians, the black whiskers represent the 99.3% confidence interval, while red crosses represent outliers. The shaded background helps to visually separate the results obtained with the two methods.

A very different outcome was instead observed when making one-second acquisitions at 2 kHz over different days. This experiment was carried out to investigate the reliability of the two methods for long-term installments where baseline recalibration could be a costly procedure if frequently required. Measurements were conducted around midday, with an interval of approximately 30 min between methods, which was more than enough time for the laser system to be properly thermalized (due to the different laser working temperature in the two methods). Figure 5 shows the result of such an experiment over the course of 24 days, where the recorded temperature at the time of the experiments spanned from 23.3 to 26.2 °C. A drastic improvement can be seen when switching to three-wavelength TDLAS, as the maximum absolute relative difference in concentration between subsequent measurements dropped from two-wavelength TDLAS’s 1.4% (mean 0.6 ± 0.1%) to 0.5% (mean 0.14 ± 0.04%). Note that, as expected from the considerations made on baseline instability, the repeatability of two-wavelength TDLAS over such a long period of time is highly degraded with respect to the one shown in [13], which involved measurements repeated every two minutes. On the other hand, the repeatability of three-wavelength TDLAS is compatible (*p* = 0.054) with the one observed over the two-minute timescale, suggesting that linear baseline corrections allow us to preserve the short-term repeatability over the span of several days/weeks. This result clearly indicates that three-wavelength TDLAS is the more robust method over extended periods of time and is thus a more viable solution for applications that involve permanent installations, remarkably without compromising the short-term capabilities of two-wavelength TDLAS.

The mismatch between the measurements made with two-wavelength TDLAS and three-wavelength TDLAS can be explained by the long-term instability of the two-wavelength approach combined with the initial calibration of the three-wavelength method.

**Figure 6:**
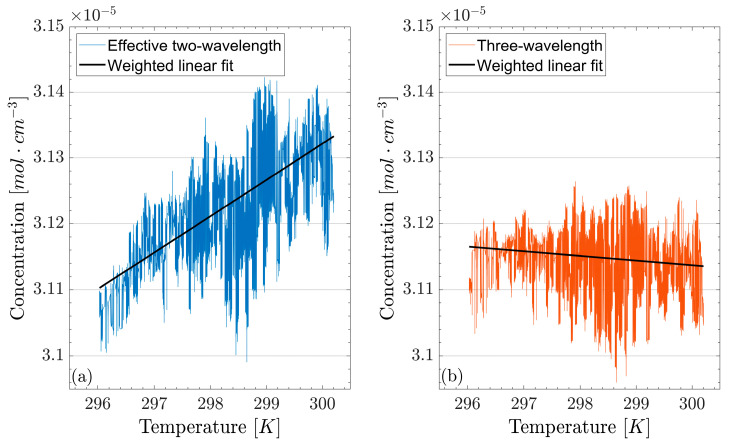
Concentration measured with (**a**) effective two-wavelength TDLAS and (**b**) three-wavelength TDLAS while changing the temperature of the beamsplitter with a soldering pen. A much stronger correlation is observed when using two wavelengths only, whilst the addition of the third symmetrically placed wavelength reduces it by a factor of 7.9 ± 0.2. The weighted linear fit used the inverse of the variances at the various temperatures as weights, calculated over 0.02 K bins.

To isolate the effect of the analytical model on the system’s stability, the results obtained with the three-wavelength technique, where the $\lambda_{1}$ and $\lambda_{2}$ data are averaged, are compared to the concentration values calculated using only one of the two, for example, $\lambda_{2}$. We refer to the latter configuration as “effective two-wavelength TDLAS”. A soldering pen was put in the proximity of the beamsplitter, forcing a change in its optical properties through a temperature-induced alteration of its refractive index. Doing this caused the baseline to vary over time, and we investigated the correlation between temperature and concentration measurements made using three-wavelength TDLAS and the newly defined effective two-wavelength TDLAS. Due to the length of the experiment, a factor 200 decimation was used. The result of this is shown in Figure 6, where a 7.9 ± 0.2 times weaker correlation can be observed when the third symmetrically placed wavelength is used, confirming the increased robustness of the newly proposed method against systematic errors. In principle, this architecture can be expected to be as reliable as the usual two-wavelength TDLAS approach but under much looser control of the environmental conditions, though the exact scale of such a benefit depends on the optical stability of the individual experimental configurations.

## 5. Conclusions

In this paper, we have introduced a novel three-wavelength TDLAS approach for real-time single-gas species quantification and simultaneous linear (and higher-order odd terms) baseline correction. This characteristic, coupled with the high stability of the targeted wavelengths, enabled our system to outperform the relative precision of other widespread spectroscopic techniques, such as scanned TDLAS, WMS and HPSDS, by almost one order of magnitude for comparable absorption values.

Working at 2 kHz, we observed an improvement in stability over the two-wavelength TDLAS method, optimized for short-term precision, for integration times below 100 s, despite a 7% lower data usage efficiency and suboptimal isolation of the absorption peak. Better repeatability with system restart was also achieved over 2 min intervals, with a mean absolute relative difference in concentration observed between subsequent 1 s measurements of 0.06 ± 0.01%, compared to the 0.08 ± 0.03% of two-wavelength TDLAS. The most significant improvement obtained was in long-term repeatability, where three-wavelength TDLAS outperformed its two-wavelength counterpart by approximately 4 times over the span of 24 days under ~3 °C ambient temperature variations. In detail, the repeatability range was 0.6 ± 0.1% for two-wavelength TDLAS and 0.14 ± 0.04% for three-wavelength TDLAS, compatible with the method’s short-timescale repeatability.

To isolate the effect of the proposed analytical model, a comparison with an effective two-wavelength TDLAS approach was performed. This consisted in considering only one of the two off-resonance wavelengths from the three-wavelength technique during the calculation of CO_2_ concentration values and comparing the results with the three-wavelength data. To force a change in the baseline, a soldering pen was placed near the beamsplitter to change its temperature and, thus, its transmittance. The three-wavelength TDLAS technique outperformed its counterpart with a 7.9 ± 0.2 times weaker correlation between concentration measurements and temperature.

The increased resilience against optical property changes in the setup makes the proposed three-wavelength TDLAS technique a very attractive candidate for applications where fast acquisitions need to be performed over extended periods of time. This is thanks to better stability under temperature fluctuations and the reduction in the need for baseline recalibrations, thus improving reliability and reducing the operational costs of the system.

## Data Availability

The original data presented in this study and used to calculate figures of merit are openly available at https://figshare.com/s/52bb27cacf35622209ef (accessed on 7 January 2026).

## Abbreviations

The following abbreviations are used in this manuscript:

DIAL	Differential absorption lidar
TDLAS	Tunable diode laser absorption spectroscopy
NIR	Near-infrared
FPGA	Field-programmable gate array
WMS	Wavelength modulation spectroscopy
HPSDS	Heterodyne phase-sensitive dispersion spectroscopy
